# Primary diffuse meningeal melanomatosis – a rare form of meningeal melanoma: case report

**DOI:** 10.1186/s12883-019-1460-x

**Published:** 2019-11-05

**Authors:** Tomasz Garbacz, Michał Osuchowski, Halina Bartosik-Psujek

**Affiliations:** 1 Department of Neurology, Clinical Hospital No. 2 in Rzeszow, Rzeszow, Poland; 2 Medical Faculty, University of Rzeszow, Rzeszow, Poland; 3 Department of Pathologists, Clinical Hospital No. 2 in Rzeszow, Rzeszow, Poland

**Keywords:** Meningeal, Melanomatosis, Melanoma, Leptomeninx

## Abstract

**Background:**

Meningeal melanomatosis is a rare type of central nervous system neoplasm (with incidence ranging between 3 and 5%) that develops in the course of malignant melanoma. In a small percentage of cases, meningeal melanomatosis may develop without a primary focus. It affects the leptomeninx. The clinical activity of the disease is uncharacteristic, with a number of neurological symptoms developing over weeks or months.

**Case presentation:**

A 45-year-old male patient presented with consciousness disturbance, cognitive dysfunctions, seizures and progressive paresis. None of the examinations performed, including cerebrospinal fluid examination, neuroimaging and biopsy of the leptomeninges, permitted us to establish a diagnosis during the patient’s hospital stay. The diagnosis of meningeal melanomatosis was established after an autopsy had been carried out.

**Conclusions:**

In the absence of unequivocal test results, it is also worth taking into account the primary changes in the leptomeninx, including those caused by melanoma.

## Background

Primary meningeal melanomatosis is a extreme, aggressive form of nonmetastatic invasion of the leptomeninges by malignant melanocytic cells. Melanocytes originate from the neural crest, and during the embryonic period, they can transfer to the eyes, skin, mucous membranes and leptomeninges. In exceptional cases, melanocytes may cause the growth of primary central nervous system (CNS) melanoma. There are two forms of primary CNS melanoma: solid tumours and diffuse meningeal melanomatosis. The diffuse form represents infiltrations into the subarachnoid space and the superficial parts of the brain without a solid mass. It can occur either as a local nodular infiltration or as meningitis [1]. Primary diffuse meningeal melanomatosis is extremely rare and involves a high degree of malignancy and an unfavourable prognosis. The clinical manifestation is complex and includes seizures, verbal communication disorders, symptoms and signs of increased intracranial pressure, psychiatric disturbances, cranial nerve palsies, and spinal cord compression. A simultaneous manifestation of symptoms of damage to many CNS areas is typical. There are many diseases that they can mimic primary diffuse meningeal melanomatosis, including subacute meningitis, viral encephalitis, leukaemia, lymphoma, neurosarcoidosis, metastatic carcinoma, acute disseminated encephalomyelitis, viral encephalitis and subacute meningitis [2, 3]. If such symptoms are present in patients with no history or symptoms of cancer, they can pose a severe diagnostic and therapeutic problem.

## Case presentation

**Fig. 1:**
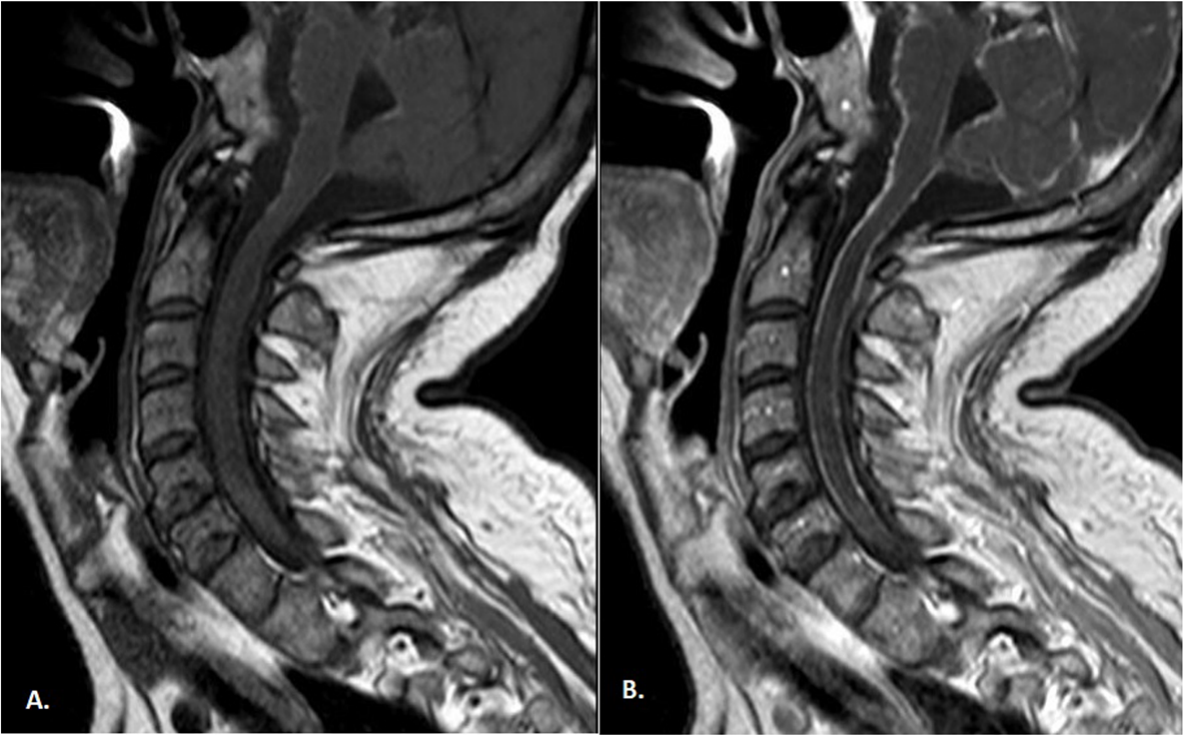
Cervical spinal cord MRI T1-weighted imaging without (**a**) and with (**b**) gadolinium contrast enhancement. Hyper-intensity on T1 was due to the paramagnetic effect of melanin, which had stable organic free radicals inside it, resulting in shortened T1 relaxation times in typical melanotic melanoma

**Fig. 2:**
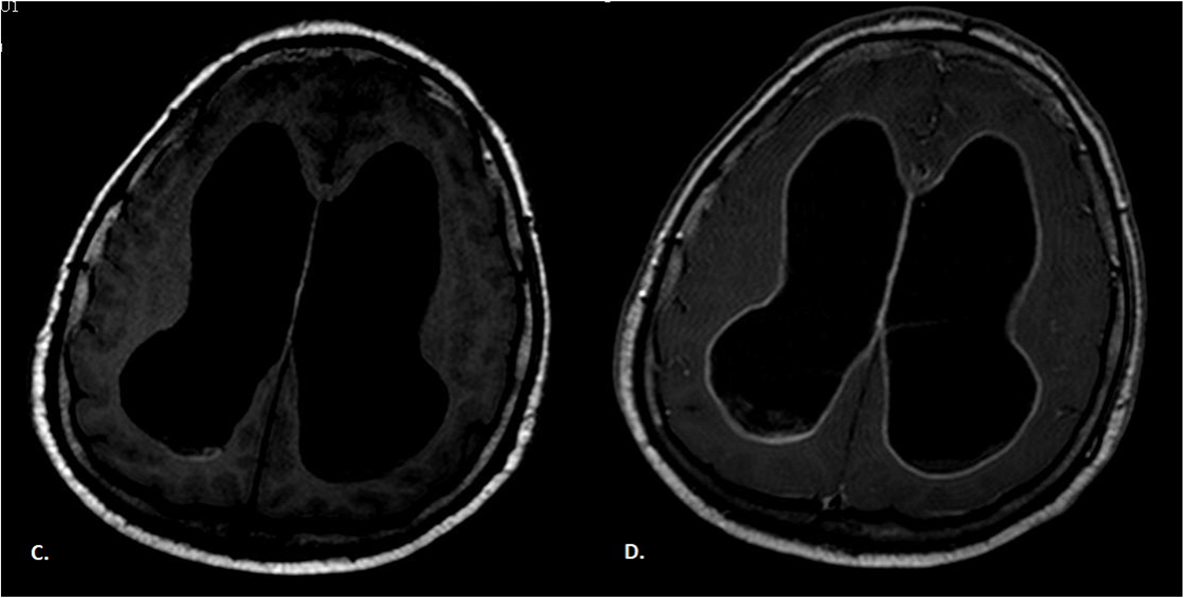
Brain MRI T1-weighted imaging without (**c**) and with (**d**) gadolinium contrast enhancement. Hydrocephalus as a consequence of disease progression. Hyper-intensity on T1 was due to the paramagnetic effect of melanin, which had stable organic free radicals inside it, resulting in shortened T1 relaxation times in typical melanotic melanoma

**Fig. 3:**
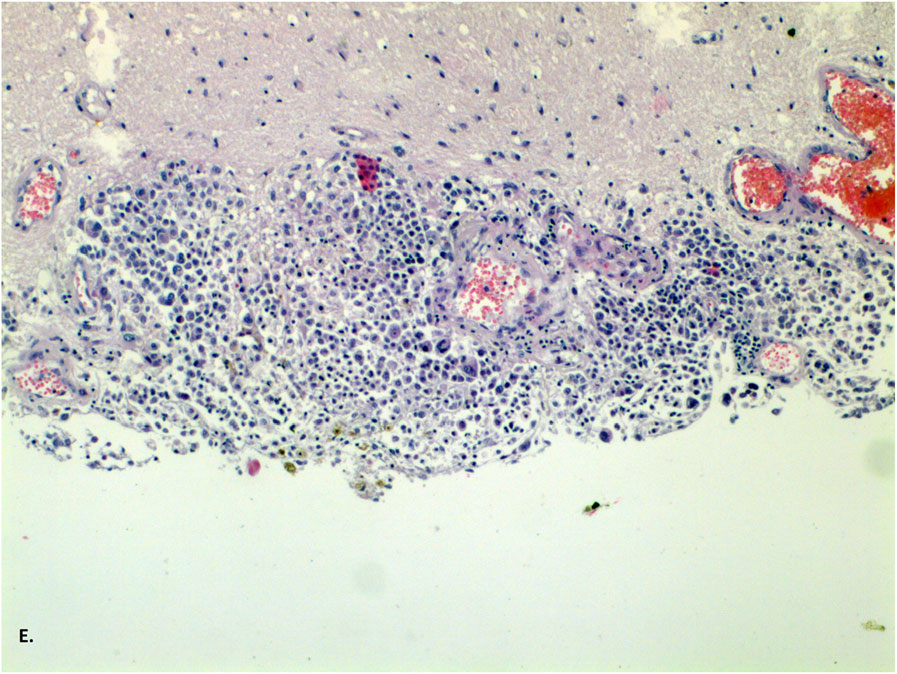
Staining with haematoxylin and eosin, enlargement 200x (**e**) Polymorphic cells infiltrating the stroma

**Fig. 4:**
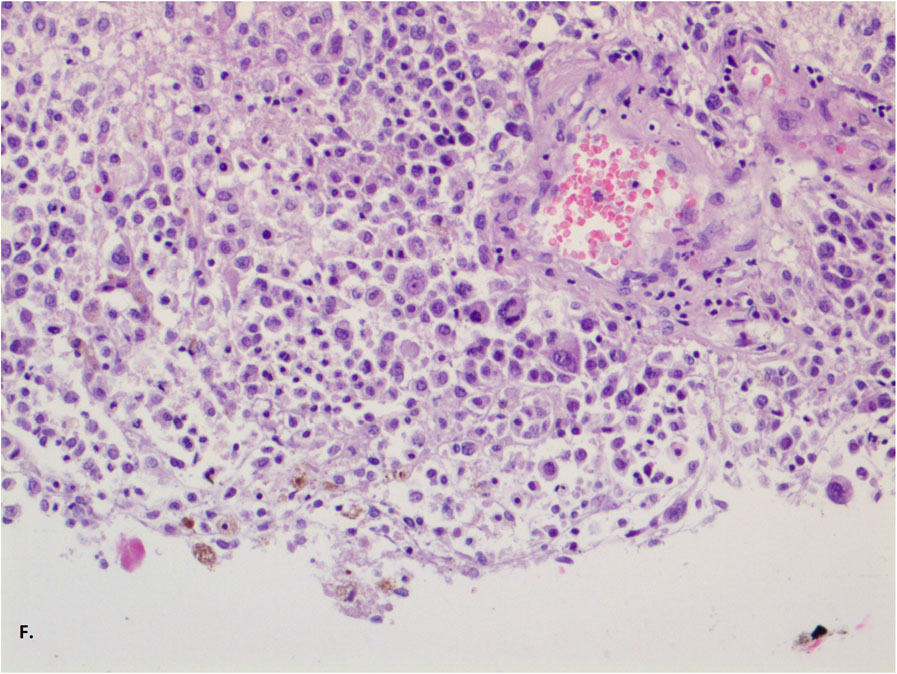
Staining of haematoxylin and eosin, enlargement 400x (**f**). There were many large, polymorphic cells with large nuclei and coarse chromatin. There was brown staining in the cytoplasm

**Fig. 5:**
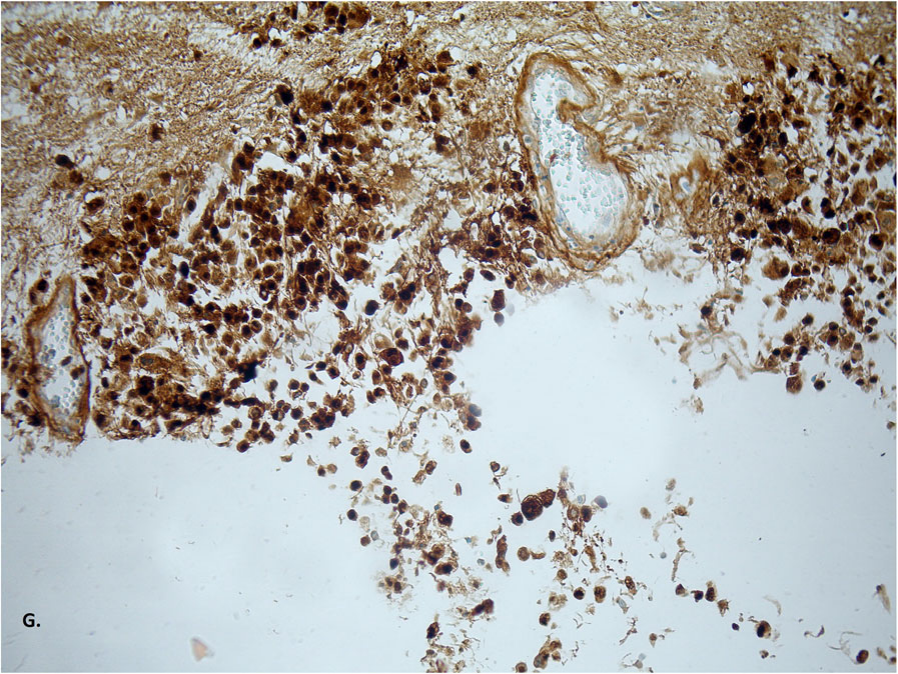
Neoplastic cells have a positive reaction in S100 staining, confirming the neuroectodermal origin; enlargement 200x (**g**)

**Fig. 6:**
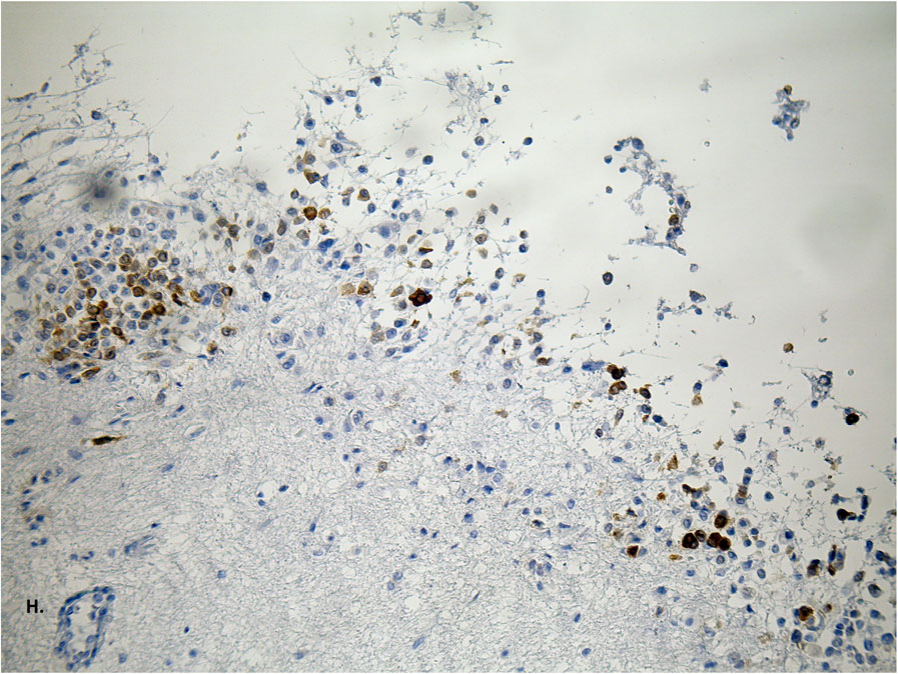
Most neoplastic cells are positive for Melano-A staining, confirming the melanocytic character of the neoplastic change; enlargement 200x (H)

A 45-year-old man was admitted to the Neurology Department at the local hospital in January 2017 due to verbal communication disturbances, nausea, vomiting, headaches, disequilibrium and a walking problem. Computer tomography (CT) of the head revealed small bilateral calcifications in the ventricles as well as reduced density of white matter. A lumbar puncture (LP) was performed. Analysis of the cerebrospinal fluid (CSF) yielded the following findings: protein 33 mg/dL, glucose 66 mg/dL, pleocytosis 70/μL – neutrophils (70%). Neuroinfection was considered, and empirical antibiotic therapy with ceftazidime was given to the patient, who was then transferred to the Department of Infectious Diseases. In the absence of the desired effect, the antibiotic was changed to ceftriaxone, and acyclovir and steroids (a daily dose of 12 mg dexamethasone for 10 days) were added. After taking these drugs, the patient reported a slight improvement and headache reduction, which were probably due to the action of the steroids. No antibody tests were carried out to determine the cause of infectious disease. The patient was discharged home. One week later, epileptic seizures appeared, and the patient was admitted to the Department of Infectious Diseases in another hospital. A head CT scan was performed, yielding results comparable to those of the previous scan. Another LP was also performed, with the following CSF findings: protein 302 mg/dL, glucose 30 mg/dL, pleocytosis 160/μL - neutrophils 80%, and negative cerebrospinal fluid culture. Blood culture, urine culture and respiratory tract culture were also negative. The liver enzyme levels were elevated, and slight hyponatraemia (up to 130 mmol/l) was observed, but the C-reactive protein (CRP) level was normal. Brain MRI revealed slight enhancement of the lining of the leptomeninx**,** mainly the ventricular ependyma, after administration of gadolinium contrast. According to the CSF findings, tuberculosis infection was the most likely cause of the symptoms, but the Bactec and QuantiFERON tests were negative. Human immunodeficiency virus (HIV), cytomegalovirus (CMV), Lyme disease, hepatitis B (HBV), and hepatitis C (HCV) infections were also excluded. A test for CMV antibodies was performed using serum and CSF samples. All of the test results were negative. The patient’s condition deteriorated; he could not walk or communicate verbally any longer, and he could only make minimal active movements with his limbs. A re-examination of CSF yielded the following findings: protein 496 mg/dL, glucose 88 mg/dL, pleocytosis 35/μL – neutrophils 80%. In May 2017, the patient was admitted to the Neurology Department in our hospital. He was conscious but unable to communicate verbally, and he had stiffness of the neck for 4 fingers, tremor of the upper limbs, and a high degree of paresis of the lower limbs. Brain MRI revealed cerebral oedema, enlarged ventricles containing blood, and strong enhancement of the ventricular lining with gadolinium contrast (Figs. 1 and 2). We noticed the presence of hydrocephalus, which was probably a consequence of disease progression. Generalized seizures intensified, and the patient’s EEG was abnormal, with mainly slow waves but without status epilepticus. A number of tests for pathogenic microorganisms were carried out repeatedly. HBV, HCV, HIV, *Francisella tularensis*, *Leptospira*, CMV, EBV, *Borrelia* and *Toxoplasma* infections were excluded. The tests for tuberculosis (Bactec and QuantiFERON) were repeated, and their results were negative as well. An LP was performed again (xanthochromia, protein 705 mg/dL, glucose 6 mg/dL, pleocytosis 90/μL – neutrophils 90%). Atypical cells characterized by a polymorphic nucleus and heterochromatin cytoplasm were found. Elevated levels of lactate dehydrogenase were found in the serum and the CSF. Because of the presence of atypical cells in the CSF, meningeal biopsy was performed, and the specimen was sent for histopathological examination. Unfortunately, the specimen did not show any abnormalities. That was probably due to a lack of noticeable changes at the biopsy site: the specimen was taken from the frontal vault, but most of the melanomatotic changes were present in the base of brain. Nevertheless, the neoplastic process was highly probable; therefore, we tried to identify the source of the tumour. The diagnostics were extended to include abdominal and chest computer tomography scans, and ophthalmoscopy of the fundus oculi as well as abdominal, thyroid and testicular ultrasound examinations. Colonoscopy was planned, but the patient’s general condition was serious. The patient was examined by specialists in cardiology, internal medicine, pulmonology, ophthalmology, infectious diseases, anaesthesiology and neurosurgery. Non-infective diseases, such as sarcoidosis or vasculitis, were taken into account. The patient’s skin was evaluated several times, but there was no suspected area requiring further analysis. Drugs were used only for symptomatic treatment. The patient’s condition worsened, and epileptic seizures were observed several times a day. Moreover, he had a fever (38–39 degrees Celsius) and heart arrhythmia. The patient died 4 weeks after admission to our hospital. The patient survived 7 months after the onset of symptoms. After his death, an autopsy was performed. There was a small amount of fuscous or brown contents under the arachnoid part of the base of the brain and the cerebellum. The ventricular system was slightly enlarged and filled with a cloudy, red-brown liquid. In the lumens of the posterior ventricles, there were soft, flabby, loosely attached beige masses measuring 3–4 cm. The lining of the ventricles was beige or honey-coloured, sometimes spreading and dull. There was a slight degree of cerebral oedema. The histopathological examination of the meninges showed infiltrations of histiocyte-like cells [CD68(−), S-100(+), Vim(+), PanCK(−), LCA(−)] containing deposits of a brown pigment in the cytoplasm, with local polymorphic features and enlarged cell nuclei (Figs. 3, 4, 5 and 6). The infiltrations were observed beneath the arachnoid membrane of the brain and the cerebellum and lined the ventricles**.** There were small points of necrotic tissue in the subependymal regions, along with congestion and cerebral oedema. There was neoplasm of the CNS, probably melanoma. This finding was supported by another histopathological examination of the meninges in a centre with a higher degree of reference and allowed us to recognize the meningeal melanomatosis. Based on the clinical examination, the results of additional tests and the autopsy, we could diagnose the patient with primary meningeal melanomatosis presenting as melanocytic meningitis. The above clinical case shows that despite extensive diagnosis and a number of tests, the diagnosis was extremely difficult to establish.

## Discussion and conclusions

We are presenting this case because of its rarity and the diagnostic difficulties that we encountered along the way. To date, the incidence of this disease has been reported to be 0.005 in 100,000 people. Meningeal melanomatosis is classified as a rare disease [4]. Therapeutic failure is mainly associated with the lack of quick diagnosis and thus with the lack of effective treatment. The symptoms of meningeal melanomatosis are uncharacteristic and occur in patients between 20 and 70 years of age (: 42 years), although a child with this disease has been described [5]. Meningeal melanomatosis affects the brain tissue in all of the cases described thus far and the spinal cord in 43% of cases. The most common symptoms include headache (46%), nausea or vomiting (37%), back or neck pain (24%) and weakness (22%). Other features include hydrocephalus, convulsions, ataxia, spinal cord cavity, cranial nerve palsies, intracranial haemorrhage and neuropsychiatric symptoms [6]. In the present case, meningeal melanomatosis affected both the brain and the spinal cord, and most of these symptoms were observed in our patient. We primarily suspected our patient to have tuberculous meningitis, but no tests confirmed it. We did not decide to start any anti-tuberculosis treatment. In a similar case, Lee et al. also considered tuberculosis and decided to put the patient on an anti-tuberculosis regimen and steroids, but they did not achieve any improvement [7]. In our patient, the CSF was tested many times, and the CSF was xanthochromic every time. The CSF showed inflammatory properties, CSF cultures were negative, and there were atypical cells, but the colour of the CSF remained unchanged. This was in contrast to Dean, who wrote that the brown colour of the CSF is suggestive of melanomatosis [8]. Using MRI, we observed hyperintensity on T1-weighted images and hypointensity on T2-weighted images of the lesions. This was because of the paramagnetic effect of melanin, which contains stable organic free radicals and shortens both the T1 and T2 relaxation times in typical melanotic melanoma [7]. On the other hand, the enhancement of the meninges with gadolinium contrast indicated a neoplastic process. Nevertheless, the diagnosis of neoplastic meningitis was suspected, and the paraclinical findings could be inconclusive. We did not perform 18F-choline PET/CT scans of the brain. Trinh et al. confirmed that an 18F-cholinethe PET/CT scan may show hypermetabolism in the affected tissue of the leptomeningeal [9, 10]. In most descriptions of meningeal melanomatosis, biopsy was recommended as a follow-up diagnosis. Our biopsy leptomeninges turned out to be nondiagnostic because of downloading probably part without changes. This was because the biopsy was performed on the frontal vault, but most of the melanomatotic changes were on the base of the brain. The fact that the diagnosis could not be established with biopsy is quite rare. Tekataş et al. repeated biopsy to confirm diagnosis [4]. Our patient was not given any chemotherapy. Dacarbazine (16–20% efficacy) combined with radiotherapy and chemotherapy has recently proven to be the most effective treatment for melanomatosis [11]. Schӓfer et al. described the first case of a patient with meningeal melanomatosis who achieved a good therapeutic effect from vemurafenib treatment [12]. Oral doses of 960 mg twice a day caused the composition of the cerebrospinal fluid to normalize, and after some time, the neoplastic cells disappeared completely. Most surprisingly, neurological deficits subsided completely under conditions where a significant clinical improvement was uncommon. Vemurafenib is a BRAF inhibitor that has been shown to be effective in patients with BRAF V600E melanoma. For local changes, the efficacy of surgical treatment was confirmed [7]. In summary, patients suffering from meningeal melanomatosis require very thorough clinical, radiological and, above all, histopathological diagnosis. Histopathological examinations should be performed in units with extensive experience. Early diagnosis gives us a chance to try the right treatment. Meningeal melanomatosis should be considered as a possible cause.

## Data Availability

The dataset supporting the conclusion of this article is included within the article.

## Abbreviations

CMV: cytomegalovirus
CSF: cerebrospinal fluid
CT: computed tomography
EBV: Epstein–Barr virus
HBV: hepatitis B virus
HCV: hepatitis C virus
HIV: human immunodeficiency virus
PET/CT: positron emission tomography–computed tomography
